# Body mass index and early outcomes after carotid endarterectomy

**DOI:** 10.1371/journal.pone.0278298

**Published:** 2022-12-20

**Authors:** Danka Vukašinović, Miloš Maksimović, Slobodan Tanasković, Jelena Marinković, Predrag Gajin, Nenad Ilijevski, Nađa Vasiljević, Đorđe Radak, Hristina Vlajinac

**Affiliations:** 1 Faculty of Medicine, Institute of Hygiene and Medical Ecology, University of Belgrade, Belgrade, Serbia; 2 Vascular Surgery Clinic, “Dedinje” Cardiovascular Institute, Belgrade, Serbia; 3 Faculty of Medicine, University of Belgrade, Belgrade, Serbia; 4 Faculty of Medicine, Institute of Medical Statistics and Informatics, University of Belgrade, Belgrade, Serbia; 5 Faculty of Medicine, Institute of Epidemiology, University of Belgrade, Belgrade, Serbia; IRCCS Policlinico S.Donato, ITALY

## Abstract

As the existing data on the correlation of adiposity with adverse outcomes of carotid endarterectomy (CEA) are inconsistent, the aim of the present study is to examine the correlation of an increased body mass index with 30-day complications after carotid endarterectomy. The cohort study comprises 1586 CEAs, performed at the Clinic for Vascular Surgery in Belgrade, from 2012–2017. Out of them, 550 CEAs were performed in patients with normal body mass index (18.5–24.9), 750 in overweight (25.0–29.9), and 286 in obese (≥30) patients. The association of overweight and obesity with early outcomes of carotid endarterectomy was assessed using univariate and multivariate logistic regression analysis. Overweight patients, in whom CEAs were performed, were significantly more frequently males, compared to normal weight patients–Odds Ratio (OR) 1.51 (95% confidence interval– 1.19–1.89). Moreover, overweight patients significantly more frequently had non-insulin-dependent diabetes mellitus—OR 1.44 (1.09–1.90), and more frequently used ACEI in hospital discharge therapy—OR 1.41 (1.07–1.84) than normal weight patients. Additionally, the CEAs in them were less frequently followed by bleedings—OR 0.37 (0.16–0.83). Compared to normal weight patients, obese patients were significantly younger—OR 0.98 (0.96–0.99), and with insulin-dependent and non-insulin-dependent diabetes mellitus—OR 1.83 (1.09–3.06) and OR 2.13 (1.50–3.01) respectively. They also more frequently had increased triglyceride levels–OR 1.36 (1.01–1.83), and more frequently used oral anticoagulants in therapy before the surgery–OR 2.16 (1.11–4.19). According to the results obtained, overweight and obesity were not associated with an increased death rate, transient ischemic attack (TIA), stroke, myocardial infarction, or with minor complications, and the need for reoperation after carotid endarterectomy. The only exception was bleeding, which was significantly less frequent after CEA in overweight compared to normal weight patients.

## Introduction

According to the World Health Organization (WHO), stroke is the second leading cause of death in the world, right after ischemic heart disease [1]. Significant extracranial carotid artery stenosis might be one of the reasons for ischemic neurological conditions and stroke [2]. Carotid endarterectomy (CEA) is considered the gold standard in the treatment of extracranial carotid disease if the incidence of adverse early and late outcomes does not exceed acceptable values according to the existing vascular guidelines [3]. However, a higher frequency of adverse outcomes reduces the benefit of CEA, especially in asymptomatic patients. Therefore, it is necessary to identify patients with an increased risk of postoperative complications, which would enable adequate selection of those who will undergo surgery [3]. The available literature describes that risk factors for postoperative complications after CEA are most often related to age, sex, body mass index (BMI), the presence of other diseases, especially diabetes, the degree of stenosis and plaque characteristics, type of surgery, and anesthesia [3–7].

The prevalence of obesity is growing worldwide and, according to the WHO, more than 50% of adults in Europe are overweight and 20% are obese [8]. In Serbia, according to the latest health survey from 2019, 36.3% of adults are overweight, and 20.8% are obese [9]. Obesity is considered a risk factor for cardiovascular and cerebrovascular diseases, type 2 diabetes mellitus, and other diseases. Data on the correlation of obesity with CEA outcomes are inconsistent. According to the results of some studies, obesity is not associated with the occurrence of stroke and death after CEA, but there are also studies in which mortality was higher in obese people in the first 30 days after the intervention [10]. Some researchers have even found that mortality after stroke is significantly lower in obese individuals [11], which would correspond to the so-called “obesity paradox” observed in patients with acute myocardial infarction.

The aim of the present study is to examine the association of an increased BMI (overweight and obesity) with 30-day adverse outcomes after CEA.

## Materials and methods

This research is a cohort study. It comprises 1533 patients in whom 1597 CEAs were performed at the Clinic for Vascular Surgery of the Institute for Cardiovascular Diseases "Dedinje" in Belgrade, from 1.1.2012. to 31.12.2017. The following patients were excluded from the study: patients with CEA and simultaneous coronary artery bypass grafting, patients who previously had massive cerebrovascular insult (CVI) and severe neurological damage, patients with severe renal insufficiency, as well as patients with severe cardiac comorbidity.

Before considering surgery for carotid stenosis, all patients underwent duplex ultrasound of the carotid and other neck arteries, confirmed by MSCT (Multi-Slice Computed Tomography) angiography. The eversion CEA was performed in all patients, except in 7 (seven) participants in whom graft was inserted, too. All patients underwent CEA under general anesthesia.

Body mass index (BMI) was calculated as weight in kg divided by height in m^2^. It was categorized as underweight (BMI < 18.5), normal weight (BMI = 18.5–24.9), overweight (BMI = 25.0–29.9), class I obese (BMI = 30.0–34.9), class II obese (BMI = 35.0–39.9) and class III obese (BMI ≥ 40) [12].

The following variables were analyzed: sex, age, body height, body weight, smoking habits, personal history of diseases (hypertension, hyperlipoproteinemia, diabetes mellitus, myocardial infarction, congestive heart failure, peripheral arterial disease), laboratory findings (triglycerides and total cholesterol), family history of cardiovascular disease, symptoms of carotid disease, degree of stenosis, type of plaque and therapy used. Operative parameters included type of surgery (elective / urgent) and clamp duration.

The patients were monitored for the first 30 postoperative days and complications were registered as major or minor. Stroke, transient ischemic attack (TIA), myocardial infarction, and total mortality were considered major complications. Stroke was defined as the occurrence of an acute neurological event with focal symptoms and signs lasting longer than 24 hours. TIA was considered a new neurologic deficit lasting less than 24 hours with complete recovery. The diagnosis of myocardial infarction was established by a cardiologist, in patients with an increase in cardio-specific enzymes and electrocardiographic changes or symptoms consistent with myocardial ischemia. Minor complications included: bleeding, respiratory, urological, and cardiac complications (postoperative hypertension and arrhythmia, cardiac arrest), cranial nerve lesion, and the need for reoperation.

The study protocol was approved by the Ethics Committee of the Faculty of Medicine, University of Belgrade (No 1322/XII-1). Informed consent was not required.

### Statistical analysis

Categorical variables were presented as numbers and percentages, while continuous variables were presented as means and standard deviations. CEAs in overweight and obese patients were compared separately with CEAs in normal weight patients. Differences between compared groups were analyzed using univariate and multivariate logistic regression analysis. Variables associated with overweight or obesity at a level ≤ 0.10, according to univariate analysis, were included in the multivariate analysis. The selection method was backward Wald. All *p* values are based on a two-tailed test, and *P* < 0.05 was considered significant. Statistical Package for Social Sciences (SPSS), version 23 was used for the analysis.

## Results

Out of 1597 CEAs, 11 (0.7%) were performed in underweight patients, 550 (34.4%) in those with normal weight, 750 (47.0%) in overweight, 244 (15.3%) in class I obese, 36 (2.3%) in class II obese, and 6 (0.4) in class III obese patients. Due to the small number of underweight patients, they were excluded from further analysis, and, for the same reason, all classes of obese patients were taken together.

In comparison with normal weight patients, overweight patients were significantly more frequently males (*p* < 0.001), they more frequently had an aortocoronary bypass (*p* < 0.05), aneurysmatic disease (*p* ≤ 0.10), and non-insulin-dependent diabetes mellitus (*p* < 0.01) in their personal history. Obese, compared to normal weight patients, were significantly younger (*p* < 0.05), and, they more frequently had a myocardial infarction (*p* < 0.05), aortocoronary bypass (*p* ≤ 0.10), and both insulin-dependent and non-insulin-dependent diabetes mellitus (*p* ≤ 0.10 and *p* < 0.001 respectively) in their personal history. They also more frequently used oral anticoagulant therapy (*p <* 0.05) and had increased triglycerides (*p* <0.01)–Table 1.

**Table 1:** Demographic characteristics, smoking, personal history, therapy, laboratory values on admission and family history of cardiovascular diseases, by body mass index *.

Body mass index Variable	Normal weight 18.5–24.9 kg/m^2^ N = 550 (34.7%)	Overweight 25.0–29.9 kg/m^2^ N = 750 (47.3%)	Obese ≥ 30 kg/m^2^ N = 286 (18.0%)
Age, y, mean ± SD	68.83 ± 8.21	68.67 ± 7.38	**67.46 ± 8.01** ^**b**^
Gender–Male, n (%)	309 (56.2)	**495 (66.0)** ^**a**^	155 (54.2)
Smoking, n (%)	274 (49.8)	349 (46.5)	128 (44.7)
Personal history, n (%)			
Myocardial infarction	35 (6.4)	50 (6.7)	**30 (10.5)** ^**b**^
PCI	32 (5.8)	53 (7.1)	17 (5.9)
ACB	33 (6.0)	**69 (9.2)** ^**b**^	**26 (9.1)** ^**c**^
Chronic heart failure	10 (1.8)	12 (1.6)	4 (1.4)
Peripheral arterial disease	83 (15.1)	122 (16.3)	44 (15.4)
Aneurysmatic disease	11 (2.0)	**29 (3.9)** ^**c**^	6 (2.1)
Hyperlipoproteinemia	489 (88.9)	672 (89.6)	255 (89.2)
Hypertension	514 (93.4)	694 (92.5)	274 (95.8)
Diabetes mellitus–insulin dependent	38 (6.9)	56 (7.5)	**31 (10.8)** ^**c**^
Diabetes mellitus–non- insulin dependent	98 (17.8)	**180 (24.0)** ^**d**^	**90 (31.5)** ^**a**^
Therapy, n (%)			
Aspirin	438 (79.6)	613 (81.7)	239 (83.6)
Clopidogrel	154 (28.0)	211 (28.1)	72 (25.2)
OAC	19 (3.4)	33 (4.4)	**20 (7.0)** ^**b**^
ACEI	393 (71.4)	538 (71.7)	216 (75.5)
β blockers	251 (45.6)	347 (46.3)	139 (48.6)
Statin	355 (64.5)	485 (64.7)	180 (62.9)
Laboratory values on admission, n (%)			
Cholesterol ≥ 5.2 mmol/L	273 (49.6)	351 (46.8)	134 (46.8)
Triglycerides ≥ 1.7 mmol/L	270 (49.1)	395 (52.7)	**170 (59.4)** ^**d**^
Family history of CVD	239 (43.4)	347 (46.3)	138 (48.2)

PCI–Percutaneous coronary intervention; ACB–Aortocoronary bypass; OAC—oral anticoagulant; ACEI—angiotensin-converting enzyme inhibitors; CVD–cardiovascular disease.

* According to univariate logistic regression analysis. *P* values for comparison between overweight and normal weight groups and for comparison between obese and normal weight groups:

^a^ *p* < 0.001:

^b^ *p* < 0.05:

^c^ *p* ≤ 0.10:

^d^ *p* < 0.01.

Compared groups did not significantly differ neither in the characteristics of the carotid disease (presence of symptoms and complicated plaque, and degree of ipsilateral and contralateral stenosis), nor in the type of endarterectomy (elective / urgent) and clamp duration (Table 2). The significant difference was only found in hospital discharge therapy. In comparison with normal weight, overweight patients more frequently used angiotensin-converting enzyme inhibitors (*p* < 0.05), and obese patients more frequently used oral anticoagulants (*p* ≤ 0.10).

**Table 2:** Characteristics of carotid disease, operative data, and hospital discharge therapy by body mass index*.

Body mass index Variable	Normal weight 18.5–24.9 kg/m^2^ N = 550 (34.7%)	Overweight 25.0–29.9 kg/m^2^ N = 750 (47.3%)	Obese ≥ 30 kg/m^2^ N = 286 (18.0%)
Characteristics of carotid disease, n (%)			
Symptomatic	183 (33.3)	278 (37.1)	98 (34.3)
Complicated plaque	79 (14.4)	126 (16.8)	37 (12.9)
Ipsilateral stenosis, n (%)			
50–69%	82 (14.9)	112 (14.9)	48 (16.8)
70–89%	304 (55.3)	424 (56.5)	140 (48.9)
90–99%	162 (29.4)	212 (28.3)	95 (33.2)
Contralateral stenosis, n (%)			
50–69%	95 (17.3)	122 (16.3)	38 (13.3)
70–89%	57 (10.4)	73 (9.7)	26 (9.1)
90–99%	30 (5.4)	29 (3.9)	8 (2.8)
100%	38 (6.9)	61(8.1)	34 (11.9)
Operative data, n (%)			
Urgent endarterectomy	7 (1.3)	7 (0.9)	2 (0.7)
Clamp duration <10 min 10–15 min >15 min	96 (17.7) 345 (63.5) 102 (18.8)	140 (18.9) 475 (64.0) 127 (17.1)	63 (22.0) 175 (61.2) 48 (16.8)
Hospital discharge therapy, n (%)			
Aspirin	535 (97.3)	722 (96.3)	279 (97.5)
Clopidogrel	385 (70.0)	528 (70.4)	190 (66.4)
OAK	19 (3.4)	31 (4.1)	**18 (6.3)** ^**b**^
ACEI	412 (74.9)	**606 (80.8)** ^**a**^	221 (77.3)
β blockers	290 (52.7)	417 (55.6)	165 (57.7)
Statin (dose) 0 10mg 20mg 40mg	16 (2.9) 25 (4.5) 503 (91.5) 6 (1.1)	31 (4.1) 32 (4.3) 684 (91.2) 3 (0.4)	10 (3.5) 7 (2.4) 266 (93.0) 3 (1.1)

OAC—oral anticoagulant; ACEI—angiotensin-converting enzyme inhibitors.

* According to univariate logistic regression analysis. *P* values for comparison between overweight and normal weight groups and for comparison between obese and normal weight groups:

^**a**^ *p* < 0.05

^**b**^ *p* ≤ 0.10.

Perioperative (30-day) outcomes of CEA are presented in Table 3. Altogether, myocardial infarction occurred after 3 (0.2%), stroke and TIA after 45 (2.8%), and death after 7 (0.4%) of all performed CEAs. Stroke occurred after 34 (2.1%) CEAs, and it was ipsilateral after 29 (1.8%) CEAs. Of other complications, bleedings appeared after 31 (1.9%), respiratory complications after 7 (0.4%), urological after 5 (0.3%), cardiac after 11 (0.7%) and cranial nerve lesions after 14 (0.9%) of all CEAs. Reoperation was necessary in 53 (3,3%) cases. Perioperative (30-day) outcomes did not significantly differ between compared groups. The only exception were bleedings, significantly less frequent (*p* < 0.05) in overweight, compared to normal weight patients. Bleedings were diagnosed based on increased drainage and the occurrence of hematoma. There were no surgical site infections after any of the observed CEAs.

**Table 3:** Perioperative (30-day) outcomes*.

Body mass index Variable	Normal weight 18.5–24.9 kg/m^2^ N = 550 (34.7%)	Overweight 25.0–29.9 kg/m^2^ N = 750 (47.3%)	Obese ≥ 30 kg/m^2^ N = 286 (18.0%)
Complication and reoperation, n (%)			
Myocardial infarction	0	3 (0.4)	0
TIA and stroke	14 (2.5)	23 (3.1)	8 (2.8)
Death	2 (0.4)	3 (0.4)	2 (0.7)
Bleedings	17 (3.1)	**10 (1.3)** ^**a**^	4 (1.4)
Respiratory	1 (0.2)	4 (0.5)	2 (0.7)
Urological	1 (0.2)	3 (0.4)	1 (0.3)
Cardiac	3 (0.5)	4 (0.5)	4 (1.4)
Cranial nerve lesion	4 (0.7)	7 (0.9)	3 (1.0)
Reoperation	23 (4.2)	21 (2.8)	9 (3.1)

* According to univariate logistic regression analysis. *P* values for comparison between overweight and normal weight groups and for comparison between obese and normal weight groups:

^**a**^ *p* < 0.05.

Results of multivariate regression analysis are presented in Table 4. Compared to normal weight patients in whom CEAs were performed, overweight patients were significantly more frequently males (*p* < 0.001), with non-insulin-dependent diabetes mellitus (*p* = 0.010), and more frequent use of ACEI in hospital discharge therapy (*p* = 0.012). Moreover, CEAs in them were less frequently followed by bleedings (*p* = 0.017). Compared to normal weight patients, obese patients were significantly younger (*p* = 0.043), and with insulin-dependent and non-insulin-dependent diabetes mellitus (*p* = 0.021 and *p =* 0.000). They also more frequently had increased triglyceride levels (*p* = 0.040), and more frequently used oral anticoagulants in therapy before the surgery (*p* = 0.023).

**Table 4:** Significant differences between compared BMI groups according to multivariate logistic regression analysis.

Variable	Odds ratio	95% Confidence interval	*P* value
Overweight vs. normal weight:			
Gender–males vs. females	1.51	1.19–1.89	0.000
Non-insulin-dependent Diabetes Mellitus	1.44	1.09–1.90	0.010
Bleedings	0.37	0.16–0.83	0.017
ACEI in hospital discharge therapy	1.41	1.07–1.84	0.012
Obese vs. normal weight:			
Age	0.98	0.96–0.99	0.043
Insulin-dependent Diabetes Mellitus	1.83	1.09–3.06	0.021
Non-insulin-dependent Diabetes Mellitus	2.13	1.50–3.01	0.000
Triglyceride level > 1.7 mmol/L	1.36	1.01–1.83	0.043
OAC therapy before operation	2.16	1.11–4.19	0.023

ACEI—angiotensin-converting enzyme inhibitors; OAC—oral anticoagulants.

## Discussion

In the present study, the frequency of early complications after CEAs did not significantly differ between those performed in overweight and obese patients compared separately to CEAs in normal weight patients. The only exception were bleedings, significantly less frequent in overweight patients, compared to CEAs in patients with normal weight.

As already stated, obesity is considered a risk factor for many diseases, including cardiovascular and cerebrovascular diseases [13–16]. It is also found that the risk of perioperative mortality is increased among obese patients undergoing vascular surgery [17,18]. However, based on some studies on patients with cardiovascular diseases, mortality was lower in obese patients, particularly if they underwent certain cardiovascular and peripheral vascular surgical procedures [19–22]. Also, there are studies in which there were no differences in perioperative mortality, according to BMI [23,24].

Data on the association of obesity with CEA outcomes are also inconsistent. In a study by Jackson et al [25], in which 3,645 CEAs were analyzed, obesity was an independent risk factor for death and cardiac complications (myocardial infarction and cardiac arrest) after CEA. Based on the suggestion from some other studies [26,27], Jackson et al postulated the possibility that obese patients might have a higher prevalence of unrecognized and untreated comorbidities than non-obese patients, which probably contributes to the association between obesity and mortality after CEA.

Similar to the results of our investigation, in a small study of 300 CEAs [28], an increased BMI did not increase perioperative or intraoperative complication rates of CEA. In a recent study [29], including 89,079 patients, on multivariate analysis, BMI status was not associated with perioperative stroke, cranial nerve injury, or surgical site infection, and overweight and obesity were also not associated with 30-day mortality. However, patients with morbid obesity (BMI ≥ 40 kg/m^2^) had higher perioperative cardiac complications. In the same study, reoperation due to bleeding differed significantly among the BMI groups–it was more frequent in underweight patients [29]. The authors postulated that this might be due to difficulties in the recognition of hematoma in obese patients as a result of neck adiposity. In our study, hematoma, as the most frequent form of bleedings, was, according to univariate analysis, significantly more frequent after CEA in normal weight patients compared to overweight and obese. But, in multivariate analysis, the difference was significant only among overweight and normal weight patients. Some authors [30,31] mentioned that bleedings could be more frequent in subjects who used combined aspirin and clopidogrel therapy before surgery. In the present study, simultaneous use of these two remedies was present in 25.4% of cases, but their distribution did not significantly differ between the observed BMI groups.

Although obesity is a risk factor for the development of cerebrovascular disease [32], in the study by Jackson et al. [33], including the cohort of 23,652 CEA, an obesity paradox was recorded. Class I obesity has been associated with a decreased risk of stroke, independent of patients’ demographics and comorbidities. There are several possible explanations for the obesity paradox [19]. The most frequently cited explanation is that the paradoxical relationship between obesity and the risk of postoperative stroke and death may be attributed to adipokines, in particular, adipokine adiponectin, released from adipose tissue, the level of which is decreased in elevated BMI. Data are showing that decreased adiponectin levels are associated with a reduced risk of cardiovascular mortality after stroke and some heart diseases [34–36]. On the contrary, in an editorial of the International Journal of Obesity, Banack and Stokes [37], it is suggested that the paradox must be met with skepticism. Namely, there is a growing body of literature that pointed to potential methodological explanations for the obesity paradox, such as misclassification bias, caused by using BMI as a measure of obesity, reverse causation, or collider stratification bias (a form of selection bias).

One of the limitations of the present study is that some relevant data (values of low density and high-density lipoproteins, creatinine, left ventricular ejection fraction and some procedural data), were not included in the study as they were not available for all patients. Also, there is a question of whether BMI is the best measure for the assessment of the association between overweight and obesity with complications and death after CEA. It seems that abdominal adipose tissue, which is related to a higher risk of death, might be higher in patients with normal weight compared to patients with BMI ≥25 kg/ m^2^ [38]. Studies conducted in general populations [38,39] showed that other measures of adiposity, such as waist circumference, waist-to-height, and waist-to-thigh ratio, or the use of the InBody Test [21,40] were more strongly associated with the risk of stroke or death than BMI. Furthermore, because of the small numbers, the present study did not include underweight patients, obesity levels II and III were not analyzed separately, and all of them could be associated with complications after CEA.

## Conclusion

Overweight and obesity are neither associated with an increased death rate, TIA, stroke, and myocardial infarction, nor with minor complications and the need for reoperation after carotid endarterectomy. The only exception was bleeding, which was significantly less frequent after CEA in overweight compared to normal weight patients.
