# Correction: Body mass index and early outcomes after carotid endarterectomy

**DOI:** 10.1371/journal.pone.0299466

**Published:** 2024-02-23

**Authors:** The PLOS ONE Staff

## Notice of republication

This article was republished on February 5, 2024, to correct an error in the author’s affiliation #1 and the eighth author’s name in the PDF file. The publisher apologizes for these errors. Please download this article again to view the correct version. The originally published, uncorrected article and the republished, corrected article are provided here for reference.

## Supporting information

S1 File Originally published, uncorrected article. (PDF)

S2 File Republished, corrected article. (PDF)
