# Supplementary material for: Correction: Body mass index and early outcomes after carotid endarterectomy
Source: PLoS One. 2024 Feb 23;19(2):e0299466. doi: 10.1371/journal.pone.0299466 (PMC10889598; doi:10.1371/journal.pone.0299466)
Supplement: S2 File — (PDF) [file pone.0299466.s002.pdf]

PLOS ONE

RESEARCH ARTICLE

Body mass index and early outcomes after carotid endarterectomy

Danka Vukašinović[1], Miloš Maksimović[1]*, Slobodan Tanasković[2,3], Jelena Marinković[4], Predrag Gajin[2,3], Nenad Ilijevski[2,3], Nađa Vasiljević[1], Đorđe Radak[2,3], Hristina Vlajinac[5]

1 Faculty of Medicine, Institute of Hygiene and Medical Ecology, University of Belgrade, Belgrade, Serbia, **2** Vascular Surgery Clinic, "Dedinje" Cardiovascular Institute, Belgrade, Serbia, **3** Faculty of Medicine, University of Belgrade, Belgrade, Serbia, **4** Faculty of Medicine, Institute of Medical Statistics and Informatics, University of Belgrade, Belgrade, Serbia, **5** Faculty of Medicine, Institute of Epidemiology, University of Belgrade, Belgrade, Serbia

* milos.maksimovic@med.bg.ac.rs

Abstract

As the existing data on the correlation of adiposity with adverse outcomes of carotid endarterectomy (CEA) are inconsistent, the aim of the present study is to examine the correlation of an increased body mass index with 30-day complications after carotid endarterectomy. The cohort study comprises 1586 CEAs, performed at the Clinic for Vascular Surgery in Belgrade, from 2012–2017. Out of them, 550 CEAs were performed in patients with normal body mass index (18.5–24.9), 750 in overweight (25.0–29.9), and 286 in obese ($\geq$30) patients. The association of overweight and obesity with early outcomes of carotid endarterectomy was assessed using univariate and multivariate logistic regression analysis. Overweight patients, in whom CEAs were performed, were significantly more frequently males, compared to normal weight patients–Odds Ratio (OR) 1.51 (95% confidence interval– 1.19–1.89). Moreover, overweight patients significantly more frequently had non-insulin-dependent diabetes mellitus—OR 1.44 (1.09–1.90), and more frequently used ACEI in hospital discharge therapy—OR 1.41 (1.07–1.84) than normal weight patients. Additionally, the CEAs in them were less frequently followed by bleedings—OR 0.37 (0.16–0.83). Compared to normal weight patients, obese patients were significantly younger—OR 0.98 (0.96–0.99), and with insulin-dependent and non-insulin-dependent diabetes mellitus—OR 1.83 (1.09–3.06) and OR 2.13 (1.50–3.01) respectively. They also more frequently had increased triglyceride levels–OR 1.36 (1.01–1.83), and more frequently used oral anticoagulants in therapy before the surgery–OR 2.16 (1.11–4.19). According to the results obtained, overweight and obesity were not associated with an increased death rate, transient ischemic attack (TIA), stroke, myocardial infarction, or with minor complications, and the need for reoperation after carotid endarterectomy. The only exception was bleeding, which was significantly less frequent after CEA in overweight compared to normal weight patients.

Check for updates

OPEN ACCESS

Citation: Vukašinović D, Maksimović M, Tanasković S, Marinković J, Gajin P, Ilijevski N, et al. (2022) Body mass index and early outcomes after carotid endarterectomy. PLoS ONE 17(12): e0278298. https://doi.org/10.1371/journal.pone.0278298

Editor: Andrea Ballotta, IRCCS Policlinico S. Donato, ITALY

Received: February 9, 2022

Accepted: November 14, 2022

Published: December 20, 2022

Copyright: © 2022 Vukašinović et al. This is an open access article distributed under the terms of the Creative Commons Attribution License, which permits unrestricted use, distribution, and reproduction in any medium, provided the original author and source are credited.

Data Availability Statement: All relevant data are within the paper and its Supporting Information files.

Funding: The authors received no specific funding for this work.

Competing interests: The authors have declared that no competing interests exist.

Introduction

According to the World Health Organization (WHO), stroke is the second leading cause of death in the world, right after ischemic heart disease [1]. Significant extracranial carotid artery stenosis might be one of the reasons for ischemic neurological conditions and stroke [2]. Carotid endarterectomy (CEA) is considered the gold standard in the treatment of extracranial carotid disease if the incidence of adverse early and late outcomes does not exceed acceptable values according to the existing vascular guidelines [3]. However, a higher frequency of adverse outcomes reduces the benefit of CEA, especially in asymptomatic patients. Therefore, it is necessary to identify patients with an increased risk of postoperative complications, which would enable adequate selection of those who will undergo surgery [3]. The available literature describes that risk factors for postoperative complications after CEA are most often related to age, sex, body mass index (BMI), the presence of other diseases, especially diabetes, the degree of stenosis and plaque characteristics, type of surgery, and anesthesia [3–7].

The prevalence of obesity is growing worldwide and, according to the WHO, more than 50% of adults in Europe are overweight and 20% are obese [8]. In Serbia, according to the latest health survey from 2019, 36.3% of adults are overweight, and 20.8% are obese [9]. Obesity is considered a risk factor for cardiovascular and cerebrovascular diseases, type 2 diabetes mellitus, and other diseases. Data on the correlation of obesity with CEA outcomes are inconsistent. According to the results of some studies, obesity is not associated with the occurrence of stroke and death after CEA, but there are also studies in which mortality was higher in obese people in the first 30 days after the intervention [10]. Some researchers have even found that mortality after stroke is significantly lower in obese individuals [11], which would correspond to the so-called "obesity paradox" observed in patients with acute myocardial infarction.

The aim of the present study is to examine the association of an increased BMI (overweight and obesity) with 30-day adverse outcomes after CEA.

Materials and methods

This research is a cohort study. It comprises 1533 patients in whom 1597 CEAs were performed at the Clinic for Vascular Surgery of the Institute for Cardiovascular Diseases "Dedinje" in Belgrade, from 1.1.2012. to 31.12.2017. The following patients were excluded from the study: patients with CEA and simultaneous coronary artery bypass grafting, patients who previously had massive cerebrovascular insult (CVI) and severe neurological damage, patients with severe renal insufficiency, as well as patients with severe cardiac comorbidity.

Before considering surgery for carotid stenosis, all patients underwent duplex ultrasound of the carotid and other neck arteries, confirmed by MSCT (Multi-Slice Computed Tomography) angiography. The eversion CEA was performed in all patients, except in 7 (seven) participants in whom graft was inserted, too. All patients underwent CEA under general anesthesia.

Body mass index (BMI) was calculated as weight in kg divided by height in m^2. It was categorized as underweight (BMI $<$ 18.5), normal weight (BMI = 18.5–24.9), overweight (BMI = 25.0–29.9), class I obese (BMI = 30.0–34.9), class II obese (BMI = 35.0–39.9) and class III obese (BMI $\geq$ 40) [12].

The following variables were analyzed: sex, age, body height, body weight, smoking habits, personal history of diseases (hypertension, hyperlipoproteinemia, diabetes mellitus, myocardial infarction, congestive heart failure, peripheral arterial disease), laboratory findings (triglycerides and total cholesterol), family history of cardiovascular disease, symptoms of carotid disease, degree of stenosis, type of plaque and therapy used. Operative parameters included type of surgery (elective / urgent) and clamp duration.

The patients were monitored for the first 30 postoperative days and complications were registered as major or minor. Stroke, transient ischemic attack (TIA), myocardial infarction, and total mortality were considered major complications. Stroke was defined as the occurrence of an acute neurological event with focal symptoms and signs lasting longer than 24 hours. TIA was considered a new neurologic deficit lasting less than 24 hours with complete recovery. The diagnosis of myocardial infarction was established by a cardiologist, in patients with an increase in cardio-specific enzymes and electrocardiographic changes or symptoms consistent with myocardial ischemia. Minor complications included: bleeding, respiratory, urological, and cardiac complications (postoperative hypertension and arrhythmia, cardiac arrest), cranial nerve lesion, and the need for reoperation.

The study protocol was approved by the Ethics Committee of the Faculty of Medicine, University of Belgrade (No 1322/XII-1). Informed consent was not required.

Statistical analysis

Categorical variables were presented as numbers and percentages, while continuous variables were presented as means and standard deviations. CEAs in overweight and obese patients were compared separately with CEAs in normal weight patients. Differences between compared groups were analyzed using univariate and multivariate logistic regression analysis. Variables associated with overweight or obesity at a level ≤ 0.10, according to univariate analysis, were included in the multivariate analysis. The selection method was backward Wald. All p values are based on a two-tailed test, and $P < 0.05$ was considered significant. Statistical Package for Social Sciences (SPSS), version 23 was used for the analysis.

Results

Out of 1597 CEAs, 11 (0.7%) were performed in underweight patients, 550 (34.4%) in those with normal weight, 750 (47.0%) in overweight, 244 (15.3%) in class I obese, 36 (2.3%) in class II obese, and 6 (0.4) in class III obese patients. Due to the small number of underweight patients, they were excluded from further analysis, and, for the same reason, all classes of obese patients were taken together.

In comparison with normal weight patients, overweight patients were significantly more frequently males ($p < 0.001$), they more frequently had an aortocoronary bypass ($p < 0.05$), aneurysmatic disease ($p \leq 0.10$), and non-insulin-dependent diabetes mellitus ($p < 0.01$) in their personal history. Obese, compared to normal weight patients, were significantly younger ($p < 0.05$), and, they more frequently had a myocardial infarction ($p < 0.05$), aortocoronary bypass ($p \leq 0.10$), and both insulin-dependent and non-insulin-dependent diabetes mellitus ($p \leq 0.10$ and $p < 0.001$ respectively) in their personal history. They also more frequently used oral anticoagulant therapy ($p < 0.05$) and had increased triglycerides ($p <0.01$)–Table 1.

Compared groups did not significantly differ neither in the characteristics of the carotid disease (presence of symptoms and complicated plaque, and degree of ipsilateral and contralateral stenosis), nor in the type of endarterectomy (elective / urgent) and clamp duration (Table 2). The significant difference was only found in hospital discharge therapy. In comparison with normal weight, overweight patients more frequently used angiotensin-converting enzyme inhibitors ($p < 0.05$), and obese patients more frequently used oral anticoagulants ($p \leq 0.10$).

Perioperative (30-day) outcomes of CEA are presented in Table 3. Altogether, myocardial infarction occurred after 3 (0.2%), stroke and TIA after 45 (2.8%), and death after 7 (0.4%) of all performed CEAs. Stroke occurred after 34 (2.1%) CEAs, and it was ipsilateral after 29 (1.8%) CEAs. Of other complications, bleedings appeared after 31 (1.9%), respiratory

Table 1. Demographic characteristics, smoking, personal history, therapy, laboratory values on admission and family history of cardiovascular diseases, by body mass index *.

Body mass index<br>Variable	Normal weight<br>18.5–24.9 kg/m^2<br>N = 550 (34.7%)	Overweight<br>25.0–29.9 kg/m^2<br>N = 750 (47.3%)	Obese<br>≥ 30 kg/m^2<br>N = 286 (18.0%)
Age, y, mean ± SD	68.83 ± 8.21	68.67 ± 7.38	**67.46 ± 8.01**[b]
Gender–Male, n (%)	309 (56.2)	**495 (66.0)**[a]	155 (54.2)
Smoking, n (%)	274 (49.8)	349 (46.5)	128 (44.7)
Personal history, n (%)			
Myocardial infarction	35 (6.4)	50 (6.7)	**30 (10.5)**[b]
PCI	32 (5.8)	53 (7.1)	17 (5.9)
ACB	33 (6.0)	**69 (9.2)** [b]	**26 (9.1)**[c]
Chronic heart failure	10 (1.8)	12 (1.6)	4 (1.4)
Peripheral arterial disease	83 (15.1)	122 (16.3)	44 (15.4)
Aneurysmatic disease	11 (2.0)	**29 (3.9)**[c]	6 (2.1)
Hyperlipoproteinemia	489 (88.9)	672 (89.6)	255 (89.2)
Hypertension	514 (93.4)	694 (92.5)	274 (95.8)
Diabetes mellitus–insulin dependent	38 (6.9)	56 (7.5)	**31 (10.8)**[c]
Diabetes mellitus–non- insulin dependent	98 (17.8)	**180 (24.0)**[d]	**90 (31.5)**[a]
Therapy, n (%)			
Aspirin	438 (79.6)	613 (81.7)	239 (83.6)
Clopidogrel	154 (28.0)	211 (28.1)	72 (25.2)
OAC	19 (3.4)	33 (4.4)	**20 (7.0)**[b]
ACEI	393 (71.4)	538 (71.7)	216 (75.5)
β blockers	251 (45.6)	347 (46.3)	139 (48.6)
Statin	355 (64.5)	485 (64.7)	180 (62.9)
Laboratory values on admission, n (%)			
Cholesterol ≥ 5.2 mmol/L	273 (49.6)	351 (46.8)	134 (46.8)
Triglycerides ≥ 1.7 mmol/L	270 (49.1)	395 (52.7)	**170 (59.4)**[d]
Family history of CVD	239 (43.4)	347 (46.3)	138 (48.2)

PCI–Percutaneous coronary intervention; ACB–Aortocoronary bypass; OAC—oral anticoagulant; ACEI—angiotensin-converting enzyme inhibitors; CVD–cardiovascular disease.

* According to univariate logistic regression analysis. *P* values for comparison between overweight and normal weight groups and for comparison between obese and normal weight groups:

[a] $p < 0.001$:

[b] $p < 0.05$:

[c] $p \leq 0.10$:

[d] $p < 0.01$.

https://doi.org/10.1371/journal.pone.0278298.t001

complications after 7 (0.4%), urological after 5 (0.3%), cardiac after 11 (0.7%) and cranial nerve lesions after 14 (0.9%) of all CEAs. Reoperation was necessary in 53 (3,3%) cases. Perioperative (30-day) outcomes did not significantly differ between compared groups. The only exception were bleedings, significantly less frequent ($p < 0.05$) in overweight, compared to normal weight patients. Bleedings were diagnosed based on increased drainage and the occurrence of hematoma. There were no surgical site infections after any of the observed CEAs.

Results of multivariate regression analysis are presented in Table 4. Compared to normal weight patients in whom CEAs were performed, overweight patients were significantly more frequently males ($p < 0.001$), with non-insulin-dependent diabetes mellitus ($p = 0.010$), and more frequent use of ACEI in hospital discharge therapy ($p = 0.012$). Moreover, CEAs in them

Table 2. Characteristics of carotid disease, operative data, and hospital discharge therapy by body mass index*.

Body mass index<br>Variable	Normal weight<br>18.5–24.9 kg/m^2<br>N = 550 (34.7%)	Overweight<br>25.0–29.9 kg/m^2<br>N = 750 (47.3%)	Obese<br>$\geq$ 30 kg/m^2<br>N = 286 (18.0%)
Characteristics of carotid disease, n (%)			
Symptomatic	183 (33.3)	278 (37.1)	98 (34.3)
Complicated plaque	79 (14.4)	126 (16.8)	37 (12.9)
Ipsilateral stenosis, n (%)			
50–69%	82 (14.9)	112 (14.9)	48 (16.8)
70–89%	304 (55.3)	424 (56.5)	140 (48.9)
90–99%	162 (29.4)	212 (28.3)	95 (33.2)
Contralateral stenosis, n (%)			
50–69%	95 (17.3)	122 (16.3)	38 (13.3)
70–89%	57 (10.4)	73 (9.7)	26 (9.1)
90–99%	30 (5.4)	29 (3.9)	8 (2.8)
100%	38 (6.9)	61(8.1)	34 (11.9)
Operative data, n (%)			
Urgent endarterectomy	7 (1.3)	7 (0.9)	2 (0.7)
Clamp duration<br><10 min<br>10–15 min<br>>15 min	 96 (17.7)<br>345 (63.5)<br>102 (18.8)	 140 (18.9)<br>475 (64.0)<br>127 (17.1)	 63 (22.0)<br>175 (61.2)<br>48 (16.8)
Hospital discharge therapy, n (%)			
Aspirin	535 (97.3)	722 (96.3)	279 (97.5)
Clopidogrel	385 (70.0)	528 (70.4)	190 (66.4)
OAK	19 (3.4)	31 (4.1)	**18 (6.3)**[b]
ACEI	412 (74.9)	**606 (80.8)**[a]	221 (77.3)
β blockers	290 (52.7)	417 (55.6)	165 (57.7)
Statin (dose)<br>0<br>10mg<br>20mg<br>40mg	 16 (2.9)<br>25 (4.5)<br>503 (91.5)<br>6 (1.1)	 31 (4.1)<br>32 (4.3)<br>684 (91.2)<br>3 (0.4)	 10 (3.5)<br>7 (2.4)<br>266 (93.0)<br>3 (1.1)

OAC—oral anticoagulant; ACEI—angiotensin-converting enzyme inhibitors.

* According to univariate logistic regression analysis. *P* values for comparison between overweight and normal weight groups and for comparison between obese and normal weight groups:

[a] $p < 0.05$

[b] $p \leq 0.10$.

https://doi.org/10.1371/journal.pone.0278298.t002

were less frequently followed by bleedings ($p = 0.017$). Compared to normal weight patients, obese patients were significantly younger ($p = 0.043$), and with insulin-dependent and non-insulin-dependent diabetes mellitus ($p = 0.021$ and $p = 0.000$). They also more frequently had increased triglyceride levels ($p = 0.040$), and more frequently used oral anticoagulants in therapy before the surgery ($p = 0.023$).

Discussion

In the present study, the frequency of early complications after CEAs did not significantly differ between those performed in overweight and obese patients compared separately to CEAs in normal weight patients. The only exception were bleedings, significantly less frequent in overweight patients, compared to CEAs in patients with normal weight.

Table 3. Perioperative (30-day) outcomes*.

Body mass index<br>Variable	Normal weight<br>18.5–24.9 kg/m^2<br>N = 550 (34.7%)	Overweight<br>25.0–29.9 kg/m^2<br>N = 750 (47.3%)	Obese<br>≥ 30 kg/m^2<br>N = 286 (18.0%)
Complication and reoperation, n (%)			
Myocardial infarction	0	3 (0.4)	0
TIA and stroke	14 (2.5)	23 (3.1)	8 (2.8)
Death	2 (0.4)	3 (0.4)	2 (0.7)
Bleedings	17 (3.1)	**10 (1.3)**[a]	4 (1.4)
Respiratory	1 (0.2)	4 (0.5)	2 (0.7)
Urological	1 (0.2)	3 (0.4)	1 (0.3)
Cardiac	3 (0.5)	4 (0.5)	4 (1.4)
Cranial nerve lesion	4 (0.7)	7 (0.9)	3 (1.0)
Reoperation	23 (4.2)	21 (2.8)	9 (3.1)

* According to univariate logistic regression analysis. *P* values for comparison between overweight and normal weight groups and for comparison between obese and normal weight groups:

[a] $p < 0.05$.

https://doi.org/10.1371/journal.pone.0278298.t003

As already stated, obesity is considered a risk factor for many diseases, including cardiovascular and cerebrovascular diseases [13–16]. It is also found that the risk of perioperative mortality is increased among obese patients undergoing vascular surgery [17,18]. However, based on some studies on patients with cardiovascular diseases, mortality was lower in obese patients, particularly if they underwent certain cardiovascular and peripheral vascular surgical procedures [19–22]. Also, there are studies in which there were no differences in perioperative mortality, according to BMI [23,24].

Data on the association of obesity with CEA outcomes are also inconsistent. In a study by Jackson et al [25], in which 3,645 CEAs were analyzed, obesity was an independent risk factor for death and cardiac complications (myocardial infarction and cardiac arrest) after CEA. Based on the suggestion from some other studies [26,27], Jackson et al postulated the possibility that obese patients might have a higher prevalence of unrecognized and untreated

Table 4. Significant differences between compared BMI groups according to multivariate logistic regression analysis.

Variable	Odds ratio	95% Confidence interval	*P* value
Overweight vs. normal weight:			
Gender–males vs. females	1.51	1.19–1.89	0.000
Non-insulin-dependent Diabetes Mellitus	1.44	1.09–1.90	0.010
Bleedings	0.37	0.16–0.83	0.017
ACEI in hospital discharge therapy	1.41	1.07–1.84	0.012
Obese vs. normal weight:			
Age	0.98	0.96–0.99	0.043
Insulin-dependent Diabetes Mellitus	1.83	1.09–3.06	0.021
Non-insulin-dependent Diabetes Mellitus	2.13	1.50–3.01	0.000
Triglyceride level > 1.7 mmol/L	1.36	1.01–1.83	0.043
OAC therapy before operation	2.16	1.11–4.19	0.023

ACEI—angiotensin-converting enzyme inhibitors; OAC—oral anticoagulants.

https://doi.org/10.1371/journal.pone.0278298.t004

comorbidities than non-obese patients, which probably contributes to the association between obesity and mortality after CEA.

Similar to the results of our investigation, in a small study of 300 CEAs [28], an increased BMI did not increase perioperative or intraoperative complication rates of CEA. In a recent study [29], including 89,079 patients, on multivariate analysis, BMI status was not associated with perioperative stroke, cranial nerve injury, or surgical site infection, and overweight and obesity were also not associated with 30-day mortality. However, patients with morbid obesity (BMI $\geq$ 40 kg/m^2) had higher perioperative cardiac complications. In the same study, reoperation due to bleeding differed significantly among the BMI groups–it was more frequent in underweight patients [29]. The authors postulated that this might be due to difficulties in the recognition of hematoma in obese patients as a result of neck adiposity. In our study, hematoma, as the most frequent form of bleedings, was, according to univariate analysis, significantly more frequent after CEA in normal weight patients compared to overweight and obese. But, in multivariate analysis, the difference was significant only among overweight and normal weight patients. Some authors [30,31] mentioned that bleedings could be more frequent in subjects who used combined aspirin and clopidogrel therapy before surgery. In the present study, simultaneous use of these two remedies was present in 25.4% of cases, but their distribution did not significantly differ between the observed BMI groups.

Although obesity is a risk factor for the development of cerebrovascular disease [32], in the study by Jackson et al. [33], including the cohort of 23,652 CEA, an obesity paradox was recorded. Class I obesity has been associated with a decreased risk of stroke, independent of patients' demographics and comorbidities. There are several possible explanations for the obesity paradox [19]. The most frequently cited explanation is that the paradoxical relationship between obesity and the risk of postoperative stroke and death may be attributed to adipokines, in particular, adipokine adiponectin, released from adipose tissue, the level of which is decreased in elevated BMI. Data are showing that decreased adiponectin levels are associated with a reduced risk of cardiovascular mortality after stroke and some heart diseases [34–36]. On the contrary, in an editorial of the International Journal of Obesity, Banack and Stokes [37], it is suggested that the paradox must be met with skepticism. Namely, there is a growing body of literature that pointed to potential methodological explanations for the obesity paradox, such as misclassification bias, caused by using BMI as a measure of obesity, reverse causation, or collider stratification bias (a form of selection bias).

One of the limitations of the present study is that some relevant data (values of low density and high-density lipoproteins, creatinine, left ventricular ejection fraction and some procedural data), were not included in the study as they were not available for all patients. Also, there is a question of whether BMI is the best measure for the assessment of the association between overweight and obesity with complications and death after CEA. It seems that abdominal adipose tissue, which is related to a higher risk of death, might be higher in patients with normal weight compared to patients with BMI $\geq$25 kg/ m^2 [38]. Studies conducted in general populations [38,39] showed that other measures of adiposity, such as waist circumference, waist-to-height, and waist-to-thigh ratio, or the use of the InBody Test [21,40] were more strongly associated with the risk of stroke or death than BMI. Furthermore, because of the small numbers, the present study did not include underweight patients, obesity levels II and III were not analyzed separately, and all of them could be associated with complications after CEA.

Conclusion

Overweight and obesity are neither associated with an increased death rate, TIA, stroke, and myocardial infarction, nor with minor complications and the need for reoperation after

carotid endarterectomy. The only exception was bleeding, which was significantly less frequent after CEA in overweight compared to normal weight patients.

Author Contributions

Conceptualization: Miloš Maksimović, Slobodan Tanasković.

Formal analysis: Jelena Marinković.

Investigation: Danka Vukašinović, Predrag Gajin, Nenad Ilijevski.

Methodology: Jelena Marinković, Hristina Vlajinac.

Writing – original draft: Danka Vukašinović, Hristina Vlajinac.

Writing – review & editing: Miloš Maksimović, Slobodan Tanasković, Nađa Vasiljević, Đorđe Radak, Hristina Vlajinac.

References

1. WHO. World health statistics 2017 Geneva: World Health Organization; 2017e.
2. Paraskevas KI. Carotid artery stenosis and stroke: controversies in prevention and treatment. Ann Transl Med 2021 Jul; 9(14):1200. https://doi.org/10.21037/atm-21-149 PMID: 34430641
3. Dorigo W, Pulli R, Pratesi G, Fargion A, Marek J, Innocenti AA, et al. Early and long-term results of carotid endarterectomy in diabetic patients. J Vasc Surg 2011; 53:44–52. https://doi.org/10.1016/j.jvs.2010.08.030 PMID: 21050697
4. Davidovic L, Koncar I, Dragas M, Markovic M, Ilic N, Mutavdzic P, et al. Female and Obese Patients Might Have Higher Risk from Surgical Repair of Asymptomatic Carotid Artery Stenosis. Ann Vasc Surg. 2015; 29(6):1286–92. https://doi.org/10.1016/j.avsg.2015.03.032 PMID: 26004965
5. Casana R, Malloggi C, Andrea Odero Jr, Tolva V, Bulbulia R, Halliday A, et al. Is diabetes a marker of higher risk after carotid revascularization? Experience from a single center. Diab Vasc Dis Res. 2018; 15(4):314–321.
6. Mazzaccaro D, Modafferi A, Malacrida G and Nano G. Assessment of long-term survival and stroke after carotid endarterectomy and carotid stenting in patients older than 80 years. J Vasc Surg. 2019 Aug; 70(2):522–529.
7. Katsiki N, Dimitri P. Mikhailidis DP. Diabetes and carotid artery disease: a narrative review. Ann Transl Med. 2020 Oct; 8(19):1280. https://doi.org/10.21037/atm.2019.12.153 PMID: 33178812
8. World Health Organization, 2013. Methodology and summary Country profiles on nutrition, physical activity and obesity in the 53 WHO European Region Member States. Available at: http://www.euro.who.int/en/publications/abstracts/country-profiles-on-nutrition,-physical-activity-and-obesityin–the-53-who-european-region-member-states-methodology-and-summary-2013 [accessed January 2016].
9. Milic N, Stanisavljevic D, Krstic M, editors. The 2019 Serbian National Health Survey. Belgrade: OMNIA BGD; 2021.
10. Volkers EJ, Greving JP, Hendrikse J, Algra A L, Jaap Kappelle LJ, Becquemin J-P, et al. Body mass index and outcome after revascularization for symptomatic carotid artery stenosis. Neurology. 2017 May; 88(21):2052–2060. https://doi.org/10.1212/WNL.0000000000003957 PMID: 28446644
11. Vemmos K, Ntaios G, Spengos K, Savvari P, Vemmou A, Pappa T, et al. Association between obesity and mortality after acute first-ever stroke: the obesity-stroke paradox. Stroke 2011; 42(1):30–6. https://doi.org/10.1161/STROKEAHA.110.593434 PMID: 21127299
12. World Health Organisation, 2000. Obesity: preventing and managing the global epidemic (WHO Technical Report Series 894) Geneva.
13. Ortega FB, Lavie CJ, Blair SN. Obesity and Cardiovascular Disease. Circ Res. 2016 May 27; 118(11):1752–70. https://doi.org/10.1161/CIRCRESAHA.115.306883 PMID: 27230640
14. Mandviwala T, Khalid U, Deswal A. Obesity and Cardiovascular Disease: a Risk Factor or a Risk Marker? Curr Atheroscler Rep. 2016 May; 18(5):21. https://doi.org/10.1007/s11883-016-0575-4 PMID: 26973130
15. Li Y, Yatsuya H, Iso H, Yamagishi K, Saito I, Kokubo Y, et al. Body Mass Index and Risks of Incident Ischemic Stroke Subtypes: The Japan Public Health Center-Based Prospective (JPHC) Study. J Epidemiol. 2019 Sep 5; 29(9):325–333. https://doi.org/10.2188/jea.JE20170298 PMID: 30555115

16. Shiozawa M, Kaneko H, Itoh H, Morita K, Okada A, Matsuoka S, et al. Association of Body Mass Index with Ischemic and Hemorrhagic Stroke. Nutrients. 2021 Jul 9; 13(7):2343. https://doi.org/10.3390/nu13072343 PMID: 34371853

17. Johnson ON III, Sidawy AN, Scanlon JM, Walcott R, Arora S, Macsata RA, et al. Impact of Obesity on Outcomes after Open Surgical and Endovascular Abdominal Aortic Aneurysm Repair. J Am Coll Surg. 2010 Feb; 210(2):166–77. https://doi.org/10.1016/j.jamcollsurg.2009.10.011 PMID: 20113936

18. Giles KA, Wyers MC, Pomposelli FB, Hamdan AD, Ching YA, Schermerhorn ML. The impact of body mass index on perioperative outcomes of open and endovascular abdominal aortic aneurysm repair from the National Surgical Quality Improvement Program, 2005–2007. J Vasc Surg. 2010 Dec; 52 (6):1471–7. https://doi.org/10.1016/j.jvs.2010.07.013 PMID: 20843627

19. Galyfos G, Geropapas GI, Kerasidis S, Sianou A, Sigala F, Filis K. The effect of body mass index on major outcomes after vascular surgery. J Vasc Surg. 2017 Apr; 65(4):1193–1207. https://doi.org/10.1016/j.jvs.2016.09.032 PMID: 27876519

20. Elagizi A, Kachur S, Lavie CJ, Carbone S, Pandey A, Ortega FB, et al. An Overview and Update on Obesity and the Obesity Paradox in Cardiovascular Diseases. Prog Cardiovasc Dis. 2018 Jul-Aug; 61 (2):142–150. https://doi.org/10.1016/j.pcad.2018.07.003 PMID: 29981771

21. Abou Ghayda R, Duck-Young Park D, Lee JY, Kim JY, Lee KH, Hong SH, et al. Body mass index and mortality in patients with cardiovascular disease: an umbrella review of meta-analyses. Eur Rev Med Pharmacol Sci. 2021 Jan; 25(1): 273–286. https://doi.org/10.26355/eurrev_202101_24393 PMID: 33506916

22. Amundson DE, Djurkovic S, Matwiyoff GN. The obesity paradox. Crit Care Clin. 2010 Oct; 26(4):583–96. https://doi.org/10.1016/j.ccc.2010.06.004 PMID: 20970043

23. Khandanpour N, Armon MP, Foxall R, Meyer FJ. The effects of increasing obesity on outcomes of vascular surgery. Ann Vasc Surg. 2009 May-Jun; 23(3):310–6. https://doi.org/10.1016/j.avsg.2008.05.012 PMID: 18691822

24. Forgie K, Bozso SJ, Hong Y, Norris CM, Ishaque A, Gill RS, et al. The effects of body mass index on outcomes for patients undergoing surgical aortic valve replacement. BMC Cardiovasc Disord. 2020; 20 (1):255. https://doi.org/10.1186/s12872-020-01528-8 PMID: 32471345

25. Jackson RS, Sidawy AN, Amdur RL, Macsata RA. Obesity is an independent risk factor for death and cardiac complications after carotid endarterectomy. J Am Coll Surg. 2012; 214(2):148–155. https://doi.org/10.1016/j.jamcollsurg.2011.10.017 PMID: 22192895

26. Maruthur NM, Bolen S, Brancati FL, Clark JM. Obesity and mammography: a systematic review and meta-analysis. J Gen Intern Med. 2009; 24:665–77. https://doi.org/10.1007/s11606-009-0939-3 PMID: 19277790

27. Amy NK, Aalborg A, Lyons P, Keranen L. Barriers to routine gynecological cancer screening for white and African—American obese women. Int J Obes (Lond). 2006; 30(1):147–55. https://doi.org/10.1038/sj.ijo.0803105 PMID: 16231037

28. Tyson CA, Tonelli CM, Parikh S, Alkhatib A, Zia S, Singh H, et al. BMI Does Not Influence Complication Rate After Carotid Endarterectomy. J Vasc Surg. 2017; https://doi.org/10.1016/j.jvs.2017.03.172

29. Arinze N, Farber A, Levin SR, Cheng TW, Rybin D, Siracuse JJ. The Association of Body Mass Index with Outcomes after Carotid Endarterectomy. Ann Vasc Surg. 2021 Nov; 77:7–15. https://doi.org/10.1016/j.avsg.2021.05.046 PMID: 34437970

30. Hale B, Pan W, Misselbeck TS, Lee VV, Livesay JJ. Combined clopidogrel and aspirin therapy in patients undergoing carotid endarterectomy is associated with an increased risk of postoperative bleeding. Vascular. 2013 Aug; 21(4):197–204. https://doi.org/10.1177/1708538113478763 PMID: 23518840

31. Jones DW, Goodney PP, Conrad MF, Nolan BW, Rzucidlo EM, Powell RJ, et al. Dual antiplatelet therapy reduces stroke but increases bleeding at the time of carotid endarterectomy. J Vasc Surg. 2016 May; 63(5):1262–1270. https://doi.org/10.1016/j.jvs.2015.12.020 PMID: 26947237

32. Yatsuya H, Yamagishi K, North KE, Brancati FL, Stevens J, Folsom AR, et al. Associations of obesity measures with subtypes of ischemic stroke in the ARIC study. J Epidemiol. 2010; 20(5):347–54. https://doi.org/10.2188/jea.je20090186 PMID: 20595781

33. Jackson RS, Black JH 3rd, Lum YW, Schneider EB, Freischlag JA, Perler BA, et al. Class I obesity is paradoxically associated with decreased risk of postoperative stroke after carotid endarterectomy. J Vasc Surg. 2012; 55(5):1306–12. https://doi.org/10.1016/j.jvs.2011.11.135 PMID: 22542344

34. Nagasawa H, Yokota C, Toyoda K, Ito A, Minematsu K. High level of plasma adiponectin in acute stroke patients is associated with stroke mortality. J Neurol Sci. 2011 May 15; 304(1–2):102–6. https://doi.org/10.1016/j.jns.2011.02.002 PMID: 21377692

35. Kistorp C, Faber J, Galatius S, Gustafsson F, Frystyk J, Flyvbjerg A, et al. Plasma adiponectin, body mass index, and mortality in patients with chronic heart failure. Circulation. 2005 Sep 20; 112 (12):1756–62. https://doi.org/10.1161/CIRCULATIONAHA.104.530972 PMID: 16157772

36. Lee SH, Ha JW, Kim JS, Choi EY, Park S, Kang SM, et al. Plasma adiponectin and resistin levels as predictors of mortality in patients with acute myocardial infarction: data from infarction prognosis study registry. Coron Artery Dis. 2009 Jan; 20(1):33–9. https://doi.org/10.1097/MCA.0b013e328318ecb0 PMID: 18997620

37. Banack HR, Stokes A. The "obesity paradox" may not be a paradox at all, editorial. Int J Obes. 2017; 41:1162–1163. https://doi.org/10.1038/ijo.2017.99 PMID: 28584251

38. Pischon T, Boeing H, Hoffmann K, Bergmann M, Schulze MB, Overvad K, et al. General and abdominal adiposity and risk of death in Europe. N Engl J Med. 2008; 359(20):2105–20. https://doi.org/10.1056/NEJMoa0801891 PMID: 19005195

39. Bodenant M, Kuulasmaa K, Wagner A, Kee F, Palmieri L, Ferrario MM, et al. Measures of abdominal adiposity and the risk of stroke: the Monica Risk, Genetics, Archiving and Monograph (MORGAM) study. Stroke. 2011; 42(10):2872–7. https://doi.org/10.1161/STROKEAHA.111.614099 PMID: 21836099

40. Wells JCK, Fewtrell MS. Measuring body composition. Arch Dis Child. 2006 Jul; 91(7): 612–7. https://doi.org/10.1136/adc.2005.085522 PMID: 16790722
